# Exploring the Prebiotic Potentials of Hydrolyzed Pectins: Mechanisms of Action and Gut Microbiota Modulation

**DOI:** 10.3390/nu16213689

**Published:** 2024-10-29

**Authors:** Débora Preceliano de Oliveira, Svetoslav Dimitrov Todorov, João Paulo Fabi

**Affiliations:** 1 Department of Food Science and Experimental Nutrition, School of Pharmaceutical Sciences, University of São Paulo, São Paulo 05508-000, SP, Brazil; debora.preceliano@usp.br; 2 Food Research Center (FoRC), CEPID-FAPESP (Research, Innovation and Dissemination Centers, São Paulo Research Foundation), São Paulo 05508-080, SP, Brazil; todorov@usp.br; 3 ProBacLab, Department of Food Science and Experimental Nutrition, School of Pharmaceutical Sciences, University of São Paulo, São Paulo 05508-000, SP, Brazil; 4 Food Research Center (FoRC), CEPIX-USP, University of São Paulo, São Paulo 05508-080, SP, Brazil

**Keywords:** prebiotics, pectins, gut microbiota, beneficial properties, human health

## Abstract

The intestinal microbiota is a complex ecosystem where the microbial community (including bacteria) can metabolize available substrates via metabolic pathways specific to each species, often related in symbiotic relations. As a consequence of using available substrates and microbial growth, specific beneficial metabolites can be produced. When this reflects the health benefits for the host, these substrates can be categorized as prebiotics. Given that most prebiotic candidates must have a low molecular weight to be further metabolized by the microbiota, the role in the preliminary biological pretreatment is crucial. To provide proper substrates to the intestinal microbiota, a strategy could be to decrease the complexity of polysaccharides and reduce the levels of polymerization to low molecular weight for the target molecules, driving better solubilization and the consequent metabolic use by intestinal bacteria. When high molecular weight pectin is degraded (partially depolymerized), its solubility increases, thereby improving its utilization by gut microbiota. With regards to application, prebiotics have well-documented advantages when applied as food additives, as they improve gut health and can enhance drug effects, all shown by in vitro, in vivo, and clinical trials. In this review, we aim to provide systematic evidence for the mechanisms of action and the modulation of gut microbiota by the pectin-derived oligosaccharides produced by decreasing overall molecular weight after physical and/or chemical treatments and to compare with other types of prebiotics.

## 1. Introduction

Advances in both fundamental and applied branches of biological and health sciences have led to significant progress in understanding the complexity of nutrition, diseases, the roles of intestinal microbiota, and food supplements. Links between nutritional components, the intestinal microbiota’s well-being, and the host’s health status (humans or even other animals) were studied and suggested [1]. The ancient Greek philosopher Hippocrates suggested the essential role of a balanced diet for good health [2]. Moreover, from the perspective of 21st-century knowledge, we can state that food components play an essential role in the balance of intestinal microbiota, which, on her side, modulate the health status of the host.

The colon microbiota is incredibly complex, consisting of trillions of microorganisms, including bacteria, archaea, viruses, fungi, and protozoa, all in complex and dynamic interactions. This diverse community plays a crucial role in various bodily functions, such as digestion, immune response, and mental health [3,4]. Moreover, the microbiota composition is influenced by diet, lifestyle, antibiotics and other drugs, and genetic heritage [4].

The application of biomolecular tools in microbiological research revolutionized the field. The advancement allowed for assessing the safety of new strains proposed as probiotics has improved the ability to predict their potential benefits. This was achieved by mapping known beneficial genes in the studied strains [5]. The term “prebiotics” was introduced when it was recognized that certain non-digestible carbohydrates could play a crucial role in bacterial growth, facilitating the development of beneficial microbial populations, which in turn could promote health benefits [6].

In 1995, Glenn Gibson and Marcel Roberfroid defined prebiotics as non-digestible food ingredients that positively affect the host by selectively stimulating the growth and/or activity of beneficial bacteria in the colon [7]. Early research focused on how prebiotics could enhance the growth of beneficial bacteria such as bifidobacteria and lactobacilli. Studies on different oligosaccharides, particularly those associated with breast milk, suggested that specific microbial populations are essential for the health balance of newborns [8].

Over time, the definition of prebiotics evolved. Initially, only oligosaccharides and similar substrates were considered. However, in 2016, the International Scientific Association for Probiotics and Prebiotics (ISAPP) updated the definition to include any substrate selectively utilized by host microorganisms to confer a health benefit [9]. Some of the most common prebiotics include inulin from chicory root, β-glucan from oats, and resistant starch from grains and beans. These compounds share some common characteristics, such as high dietary fiber content, resistance to digestion in the upper gastrointestinal tract (GIT), and fermentation by beneficial bacteria in the colon [10]. Higher molecular weight polysaccharides, such as native pectins, are not as quickly metabolized by the gut microbiota [11]. Their large, complex structures make their breaking down difficult for bacterial enzymes. As a result, these molecules may pass through the intestine without being fully utilized, thus limiting their prebiotic efficacy.

Moreover, one of the requirements for a molecule to be considered a prebiotic is a selective modulation of the intestinal microbiota [9] since this prebiotic’s modulation needs to be reproducible in humans. Pectin has diverse glycosidic linkages and is found in nature as large molecules, thus usually demanding an extensive mobilization of the microbiota community. This could lead to no reproducible results (statistically significant) when clinical trials are conducted with pectins [12]. To overcome these two-pointed problems, a decrease in the complexity of pectins by reducing the molecular weight can be achieved through hydrolysis, which breaks down complex pectins into smaller, more easily fermentable oligosaccharides [13].

Pectic oligosaccharides (POS), produced by hydrolysis of pectin, are promising prebiotics owing to their high performance and relatively low cost. In addition, POS from pectin-rich agro-industrial wastes and byproducts saves resources and reduces pollution [14]. Driven by the health benefits of prebiotics, the global market was valued at $6 billion in 2022 and is projected to grow to $13.8 billion by 2030 [11].

Prebiotics have been shown to improve gut health and enhance immune function, and they may even have benefits for metabolic health, associated with their role in modulating the metabolism of beneficial microorganisms [10]. Some commercial examples of prebiotics, such as inulin and fructooligosaccharides, may improve gut health by increasing the production of short-chain fatty acids (SCFA), which have anti-inflammatory properties [6]. This review aims to provide an overview of the mechanisms through which pectin-derived oligosaccharides modulate gut microbiota.

## 2. Bibliometric Analysis

**Figure 1:**
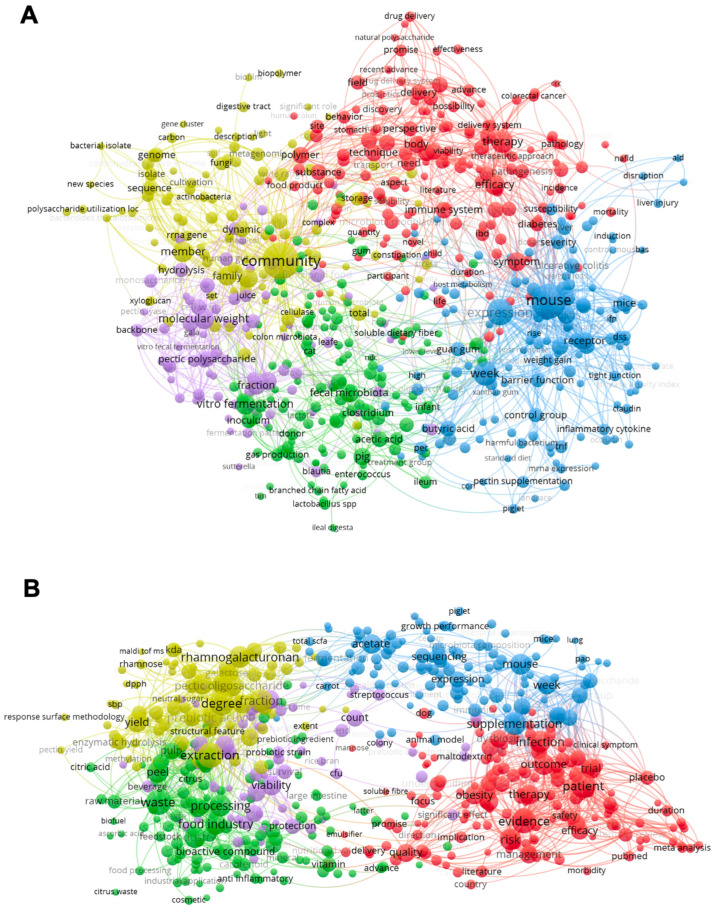

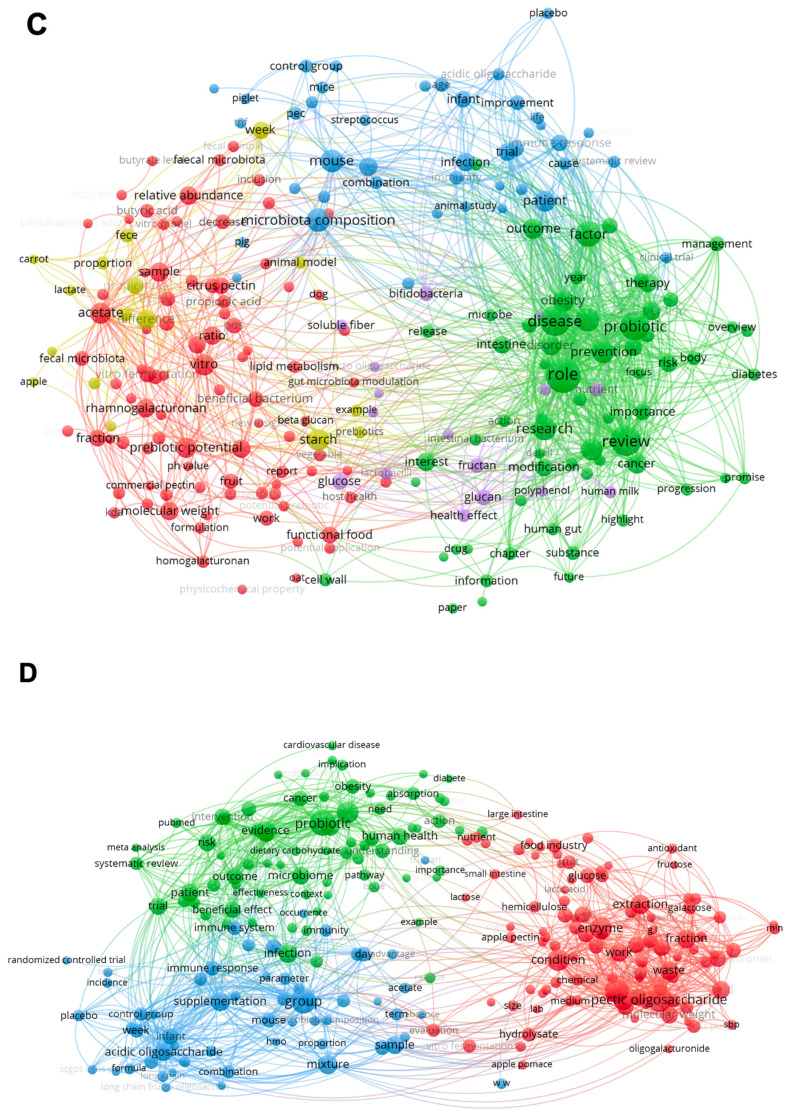
Clusters of words from articles about pectin summarized by keyword co-occurrence in VOSViewer software. (**A**) Searched keywords “microbiota” and “pectin”; (**B**) Searched keywords: “prebiotic” and “pectin”; (**C**) Searched keywords “prebiotic”, “microbiota,” and “pectin”; (**D**) Searched keywords “prebiotic”, “pectin” and “oligosaccharide”.

It is necessary to understand that pectin has been studied as a potential prebiotic in just a few years. We performed a bibliometric analysis in the Scopus database to stratify the studies regarding pectin and microbiota throughout the years by significance. The publishing range was between 2010 and 2025 using the Boolean operator “AND” between the terms; the resulting data from “Citation information” and “Abstract & keywords” were collected as a RIS file. The data clustering was performed in the VOS Viewer software (version 1.6.20 for MacOS), with a five minimum number of occurrences of a term, using the association strength method, with a layout of attraction being two and repulsion being one, with a clustering resolution of one and minimum small clusters of one, and random starts and iterations of 10 [15]. First, the search for the terms “microbiota” and “pectin” resulted in 685 articles (Figure 1A). The second search for “prebiotic” and “pectin” resulted in 600 articles (Figure 1B). It is important to highlight that some clusters were found in both analyses. The overlapped clusters were related to SCFA production, the use of animal models, in vitro fermentation, and potential therapy for non-communicable diseases (NCD). However, the most important clusters, such as the ones related to the nature of the molecules (pectin), are different between them. While the first analysis has some words related to pectin structure (molecular weight, backbone, and pectic polysaccharides), the former is more related to pectin as a prebiotic molecule (pectic oligosaccharide, prebiotic activity, and galactose), including several studies related to the use of food industry byproducts. Despite both analyses returning a fair and similar number of articles, we needed to include the words to be searched together to understand how they would connect and demonstrate an actual overlap.

The third search for “prebiotic”, “microbiota,” and “pectin” returned 223 articles, most of which were identified in the prior searches, corroborating the overlapped clusters (Figure 1C). The first cluster (green) is related to the biological effects of pectins, regardless of demonstrating prebiotic activity. The second cluster (red) is related to the pectin structures and some biological outputs (potential health benefits and metabolites produced) after in vitro fermentation, with citrus pectin as the primary type of studied source. The third cluster (blue) is related to the group of individuals where the pectins were studied, concentrated in mouse, mice, and piglet studies, with some mentions of human clinical trials. The fourth (yellow) and the fifth (purple) clusters are minor related to some biological effects and polysaccharide identities, mostly related to prebiotic potential and their outcomes, with a clear overlap with red and green clusters.

Finally, to identify if there was a correlation regarding hydrolyzed pectins (oligosaccharides) and the potential prebiotic effects, the fourth search for “prebiotic”, “pectin”, and “oligosaccharide” returned 220 articles with different clusters compared to the third search (Figure 1D). More straightforwardly, the results were divided into 3 clusters. The red one related to pectin structure focused on oligosaccharides and with hydrolyzed apple pectin as the main studied structure; the blue color related to the study conducted (in vitro, in vivo, or clinical trial); and the green cluster relates to the biological benefits outcomes. This analysis concludes that pectin is being studied as a potential prebiotic, with an increasing interest in its isolated oligosaccharides, which would justify the importance of the present review as a compilation of what the scientific community has evolved, giving new perspectives for future studies and applications. As discussed later, the prebiotic potential of hydrolyzed pectins and/or pectic oligosaccharides is more predictable than complex mixtures of high molecular weight native pectins isolated from diverse plants. In this way, the bibliometric analysis demonstrated that even though pectin is already being studied as an intestinal microbiota modulator and, to some extent, as a prebiotic, the studies regarding pectic oligosaccharides are still evolving, being crucial to conduct more clinical trials with hydrolyzed (low molecular weight) pectins and the pectic oligosaccharides.

## 3. Prebiotics: Definition, Classification and Examples

**Figure 2:**
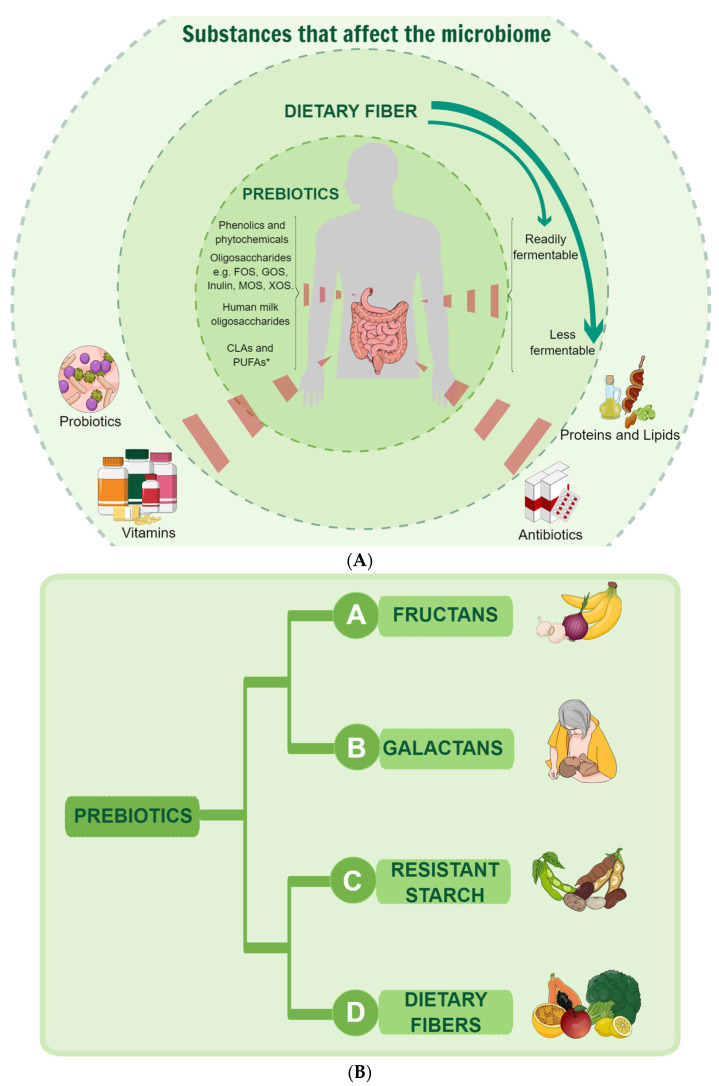
(**A**) Describing what affects the microbiome and what is considered a prebiotic with the proposed definition. Prebiotics must be selectively utilized by microorganisms and supported by sufficient evidence of health benefits for the host. Additionally, dietary prebiotics should resist degradation by the host’s enzymes. * The figure shows candidate prebiotics in that levels of evidence currently vary, with FOS and GOS being the most researched prebiotics. CLA, conjugated linoleic acid; PUFA, polyunsaturated fatty acid; FOS, fructooligosaccharides; GOS, galactooligosaccharides; MOS, mannanoligosaccharide; XOS, xylooligosaccharide (Based on Gibson et.al. [9]. (**B**) Demonstrating the principal classes of prebiotics and the examples.

Prebiotics, first defined in 1995, have been utilized to modulate host microorganisms to enhance measurable health outcomes. In 2017, the definition was updated to describe prebiotics as substances non-digestible by humans and animals but fermented by microorganisms, highlighting the specific metabolic capacities of various organisms (Figure 2A) [7,9,16]. They are classified into four principal classes (Figure 2B) based on their resistance to digestion in the upper GIT and their ability to be fermented by gut-inhabiting beneficial—and sometimes non-beneficial—bacteria.

Fructans (Group A) are one of the four principal classes of prebiotics, extensively studied in recent decades. This group includes inulin and fructooligosaccharides (FOS), known for stimulating the growth of probiotics, boosting immune response, and improving overall health [10,17,18]. Found in chicory roots, garlic, onions, and bananas, fructans are non-structural polymers of D-fructose produced by plants and microorganisms (Figure 2B) [19,20]. Structurally, they are classified into inulin, levans, and graminan. Fructans have gained attention for their non-digestible properties and potential as functional ingredients in managing chronic diseases such as hypertension, diabetes, and colon cancer [19].

Group B, galactans, includes galactooligosaccharides (GOS), derived from lactose and found in foods like vegetables [10,21,22]. GOS plays an essential role in the health and development of newborns, especially when included in infant formula. As part of the general prebiotics’ properties, GOS acts in promoting the growth of beneficial bacteria in the gut and, as a consequence, helps to improve a healthy, balanced microbiome [23,24]. Moreover, GOS is associated with supporting the development of the immune system. By promoting a balanced gut microbiota, GOS can help protect against harmful pathogens and reduce the risk of infections [25]. Additionally, GOS contributes to digestive comfort by forming softer stools and reducing the incidence of constipation, making digestion more comfortable for newborns [26].

Resistant starch (Group C) can be obtained from various grains, beans, and cooked and cooled potatoes. Resistant starches are not digested in the small intestine and reach the colon, where they are fermented. This fermentation process provides numerous benefits, including improved intestinal health, glycemic balance, lipid metabolism, and body weight regulation. While the potential role of resistant starch in reducing the risk of diet-dependent disorders such as diabetes, obesity, lipid disorders, and intestinal health issues is promising, further research is needed to reach conclusive evidence [27].

Other dietary fibers (Group D) are the category where pectin, β-glucans, and xylooligosaccharides, which are found in various fruits, vegetables, and grains can be placed [28]. These fibers are not digested in the upper GIT and reach the colon, where beneficial gut bacteria ferment them [22].

Pectins, a soluble fiber, primarily found in apples, citrus fruits, and berries, are recognized for promoting gut health by acting as prebiotics and stimulating the growth of beneficial bacteria. They also boost the production of SCFA, like butyrate, acetate, and propionate, which are vital for maintaining gut health, enhancing immune responses, and reducing pathogen’s activity [29,30].

By modulating the gut microbiota, pectins support immune function and may help prevent inflammatory bowel diseases. They also positively impact cholesterol and glucose metabolism, contributing to weight management and reducing the risk of metabolic disorders [30]. Recent findings indicate that pectins may help manage allergies by regulating gut microbiota and decreasing inflammation [31].

### Steps Required to Classify Compounds as Prebiotics

**Figure 3:**
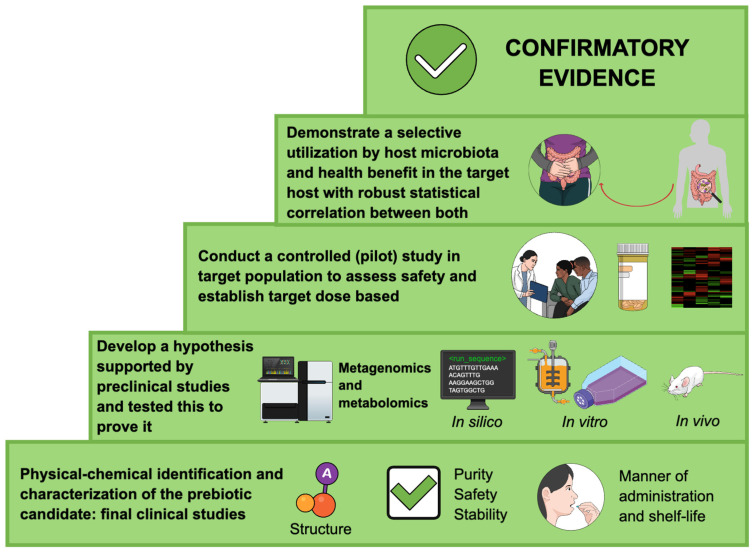
Flowchart of commonly employed steps for developing a prebiotic. Research strategies for different prebiotics might deviate as long as minimum criteria are followed (adapted from Hutkins et al. [32]).

A prebiotic must meet certain criteria, including being a substrate for microorganisms, being identified and characterized, being used by the host microbiota, demonstrating a benefit to the host’s health, and being safe for human and/or animal use. Gibson et al. [9] stated that a prebiotic must undergo randomized clinical trials to demonstrate its use and health benefits. A recent publication by ISAPP members outlined what prebiotics are and what types of research are needed to establish their biological health status (Figure 3).

The recommendation to conduct at least one clinical study in the target host, tracking both the health outcome and the selective utilization of the prebiotic by the host microbiome, remains essential. While establishing causal links between prebiotic-induced microbiota changes and health benefits is encouraged, it is not strictly mandatory. If such a causal link is demonstrated, a biologically plausible hypothesis should be supported by relevant statistical analysis explaining how microbiota modulation leads to the health effect. This evidence of selectivity may come from either compositional change in microbiota or functional readouts, with modulation ranging from narrow to broad taxonomic shifts, often reflected in altered microbial metabolic activity [32].

It is also important to highlight preclinical studies (in vitro and in vivo), although valuable for providing mechanistic insights and informing hypotheses for prospective clinical trials, are insufficient to establish a prebiotic status. Moreover, no global authority officially designates a substance as a prebiotic based only on preclinical tests. Therefore, the use of the term “prebiotic” is a judgment made by qualified scientific experts through regulatory bodies in different regions of the globe [32].

## 4. Pectin: Chemical Structures and Modification/Hydrolysis

**Figure 4:**
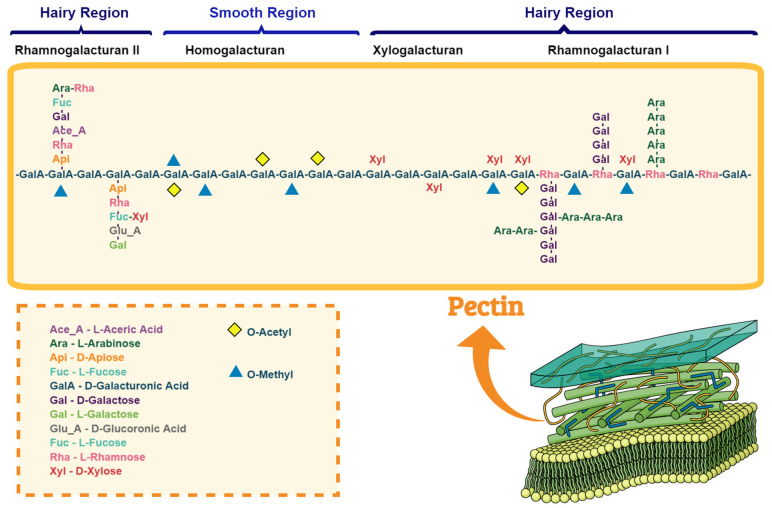
Schematic representation of pectin structure and composition, including common substitutions, such as methylation. Drawings were made using the Mind the Graph software (adapted from Roman-Benn et al. [38]).

Pectin is nature’s structurally most complex polysaccharide, a natural biopolymer that is a vital part of the cell walls and the middle lamella of higher plants [33,34]. They play an important role in cell expansion, morphogenesis, permeability, seed hydration, leaf senescence, fruit ripening, defense mechanisms, and ion binding [34,35]. The three main regions of pectin structure are homogalacturonan (HG), rhamnogalacturonan I (RG-I), and rhamnogalacturonan II (RG-II), despite their variability depending on the type of pectin (Figure 4) [33,36]. Besides what was mentioned, there is another class of pectinaceous polysaccharides called xylogalacturonan (XGA) [34].

The predominant pectic fraction is HG, primarily composed of a homopolymer of partially esterified 1-4-α-D-galactopyranuronic acid (Gal*p*A) [36]. GalA residues in HG can be methyl-esterified at the C6 position and/or acetylated at the C2 or C3 positions, which confers structural diversity on this uniform homopolymer since each residue can have up to eight different modification states [33,37]. Xylogalacturonans are homogalacturonans with a β-linked D-xylose-(1–3) at the O-3 position. A β-linked D-xylose often follows it at the O-4 position [34].

RG-I is the most branched and structurally heterogeneous fraction, based on a repeating disaccharide of Gal*p*A and rhamnose backbone [→4)-a-D-Gal*p*A-(1→2)-a-L-Rha*p*-(1→] with neutral sugar side chains, commonly known as the ‘hairy region’, with the branched-chain mainly consisting of galactose and arabinose [39]. The RG-II region consists of an HG backbone of around nine Gal*p*A residues, which may be methyl-esterified at the C-6 carboxyl groups, with varied, complex side chains of rhamnose and other neutral sugars [33,34].

The degree of methyl-esterification or degree of methylation (DM) of Gal*p*A units is used to classify pectin. DM represents a percentage that expresses the molar ratio of methyl-esters present to Gal*p*A units (including free and substituted Gal*p*A). It is the principal parameter affecting gelling, influencing surface tension and emulsion formation. Pectin is classified according to DM: low methoxy pectin (LMP), which forms gels in the presence of cations over a broad pH range, and high methoxy pectin (HMP), which forms gels in high co-solute concentration (55–75%), and acidic (pH 2.5–3.5) systems [40]. The degree of esterification (DE) plays a crucial role in pectin’s ability to form gels, being a parameter that indicates the physical, functional, and technological properties. Commercialized pectins are classified into high-esterification pectins (50% of their esterified carboxylic groups) and low-esterification pectins (50% or less of these groups are esterified) [41].

Due to the complex nature of pectin polysaccharides, as galacturonic acid, rhamnogalacturonan, and xylo-oligogalacturonan, with varying degrees of methylation and acetylation, pectin oligosaccharides (POS) exhibit diverse and source-dependent structures. These structural variations may support distinct fermentation characteristics and promote the selective growth of different intestinal microbiota species [42]. In this way, pectin has been studied as a source of POS and a potential prebiotic. One chemical characteristic that precludes pectin from being considered as prebiotics is the high molecular weight pectins are found in nature. For a substance to qualify as a prebiotic, it must selectively influence the gut microbiota [9]. This means that the modulation must be consistently replicable in human subjects. Pectin, with its varied glycosidic linkages and typically high molecular weight, often requires a substantial mobilization of the microbiota. Consequently, this can lead to inconsistent results (statistically significant) in clinical trials involving pectins [12]. POS does not have this problem. POS derived from bergamot peel through enzymatic hydrolysis, for example, has been demonstrated as a potential prebiotic [11,43,44]. Furthermore, studies have shown that POS derived from citrus (also called modified citrus pectin—MCP) and hawthorn pectin exhibits potent antimicrobial activity against food spoilage microorganisms [14].

One key factor influencing prebiotic activity is the molecular weight of POS. Prebiotic activity is most prominent when POS has a molecular weight between 1 kDa and 3 kDa, establishing this range as a critical parameter for POS quality [45]. Smaller molecules within this range can be metabolized more efficiently by gut bacteria, significantly enhancing their prebiotic potential [11].

Modification of pectin can be achieved using some techniques, among them are substitution (alkylation, amidation, thiolation, and sulfation, etc.), chain elongation (crosslinking and grafting), and depolymerization (acidic or enzymatic hydrolysis, β-elimination, and mechanical degradation) [41]. Extraction methods also generate chemical modifications in the structures, and the amount of oligosaccharides increases with excessive use [46].

Some researchers use only hot water to reduce the use of chemical reagents [46]. The high temperature facilitates the self-hydrolysis of pectin dispersed in an aqueous solution since the protons released by galacturonic acid can act as the catalyst. Besides, water serving as a reaction medium under a sub-or supercritical state could accelerate pectin self-hydrolysis, considering the presence of H_3_O^+^ species and the availability of water molecules are enhanced [13]. On the other hand, depending on factors such as high temperatures and the composition of the pectin, undesirable Maillard reactions may occur, resulting in the darkening of the pectin samples [38].

## 5. Prebiotics as Modulators of Intestinal Microbiota

The chemical characteristics of prebiotics, like molecular arrangement, size, monosaccharide number, and glycosidic linkages, are important to prospect the possible biological effects. For example, the chemical structure of fermentable fibers with prebiotic effects is directly related to the SCFA production profile: inulin is described as propionogenic, whereas resistant starches are more butyrogenic [47,48,49]. Some prebiotic compounds, like galactans, have demonstrated pharmaceutical and nutraceutical activities (e.g., galactans from prunes exhibit gastroprotective effects) [50].

The interplay between bacteria, archaea, viruses, and eukaryotic microorganisms in the host gut is necessary for maintaining overall health. Numerous studies emphasize the vital role of gut microbiota in regulating metabolism, immunity, and even behavior [51]. However, microbiota composition is not static—it is shaped by genetics, age, diet, the mode of birth, and exposure to antibiotics and drugs [16]. In these complex functional ecosystems, bacteria perform various roles, such as converting dietary carbohydrates, proteins, and some fats into metabolites that can positively or negatively impact host health. Prebiotics could be an excellent strategy to confer a health benefit. However, understanding the involved metabolic pathways in the processes is essential to implementing nutritional strategy [52].

Compounds like polyphenols (e.g., water-insoluble cocoa fraction) and polyunsaturated fatty acids (e.g., omega-3) may also have prebiotic effects [16,53]. On the other hand, substrates like vitamins, minerals, antibiotics, and bacteriophages that affect microbiota composition through mechanisms not involving selective utilization by host microorganisms are not prebiotics. To establish whether a product is genuinely prebiotic, clinical trials must demonstrate that it selectively promotes the growth of specific beneficial microorganisms, directly or indirectly (cross-feeding effect) [9].

Specific conditions in the colon may be favorable for the fermentation of non-digestible carbohydrates, including prebiotics, due to the specificity of the anaerobic environment, reasonably short transit time, and specific low pH coupled with low redox potential [50]. As a result, intestinal bacteria can bio-transform non-digestible carbohydrates and other prebiotics and generate metabolites that can be rapidly absorbed by the host intestinal epithelium [51]. Most research has focused on the biotransformation of complex carbohydrates into short-chain fatty acids (SCFA), mainly acetate (C2), propionate (C3), and butyrate (C4) [52,53]. Bacteria generate these metabolites under anaerobic conditions through the fermentation of dietary fibers, predominantly oligofructose, arabinoxylan, inulin, and pectin [54]. In addition to the commonly discussed SCFAs, prebiotics can also lead to the production of other metabolites, such as phenolic compounds and a small amount of branched-chain fatty acids (BCFAs), such as isobutyrate, valerate, and isovalerate, as well as other organic acids (e.g., lactate, succinate, and formate) [55].

The continuous breakdown of complex carbohydrates is attributed to certain abundant species within the phylum Bacteroidetes and the production of acetate. The acetate production requires substrates described as acetogenic fibers (e.g., inulin and galactooligosaccharides), and there are two possibilities of pathways: acetogenesis mediated by homoacetogenic bacteria, which can use both H_2_ and CO_2_ or carbon fixation, that produces acetate directly from CO_2_ [54].

Different pathways can produce propionate, such as *Bacteroides vulgatus* and *Bacteroides thetaiotaomicron* (considered as next-generation probiotics) using the succinate pathway (processes hexoses and pentoses using vitamin B12 to convert succinyl-Coa into propionate). In contrast, *Coprococcus catus* consumes lactate and uses the acrylate pathway (converts lactate into propionate) [54,55]. *Lachnospiraceae* uses propanediol pathway (deoxy sugars as fucose and rhamnose) [54,55,56]. A few dominant genera (e.g., *Akkermansia muciniphila*) could also produce propionate using one of the explained mechanisms [54,55].

There are four pathways to obtain butyrate using different substrates: acetyl-CoA, glutamate, lysine, and succinate. Butyrate is produced by strictly anaerobic bacteria of the clostridial groups I, III, IV, XI, XIVa, XV, and XVI, with bacteria in group XIVa and group IV being related to *Faecalibacterium prausnitzii* (abundant in humans). Members of clostridial group IX are also producers of propionate via the lactate pathway [55]. A few gut pathogens also possess butyrogenic pathways. Still, they evolved differently to produce butyrate using distinct pathways, like those for glutamate (4-aminobutyrate) and lysine, associated with releasing harmful byproducts such as ammonia [56]. The butyrate production by these bacteria is crucial for colonocytes, as it supplies them with energy and enhances epithelial oxygen consumption. This process contributes to maintaining an anaerobic environment in the gut, which is unfavorable for opportunistic aerobic pathogens such as *Salmonella* spp. and *Escherichia coli* [54].

Intestinal pH significantly influences the competition between different bacterial groups within the gut microbiota. Acidification of the medium to around pH 5.5, mainly due to fermentation, shapes the community structure and microbial activities in the colon [57]. In vitro experiments with controlled pH conditions have shown that a slightly acidic pH reduces the growth of Bacteroides relative to Firmicutes and Actinobacteria, which is explained by the lower capacity of Bacteroides compared to Firmicutes species to tolerate the presence of SCFA at pH 5.5 than at pH 6.5, limiting the propionate and stimulating butyrate production [56].

Mathematical models support these findings, indicating that butyrate production by the butyryl-CoA: acetate CoA-transferase pathway increases at acidic pH due to enhanced acetate uptake [56]. This is observed in species such as *Roseburia* spp. and *F. prausnitzii*, where net acetate uptake increases ATP yield, compensating for the effects of low pH and promoting butyrate formation. These results highlight the critical role of pH in modulating gut microbiota composition and metabolic outputs, with implications for host health [58].

As a weak acid, SCFA is ionized and requires transport to be absorbed from the colon. The following four transport mechanisms exist: passive diffusion, bicarbonate exchange, sodium-coupled, or monocarboxylate transport. Butyrate is locally absorbed and metabolized by the colonic epithelium, affecting the low concentration in portal blood. Butyrate is also involved in maintaining the integrity of the intestinal barrier, associated with promoting cell proliferation, apoptosis, tight junctions, and mucus production [59]. Acetate and propionate are drained into the portal vein, but only propionate is metabolized in the liver [60,61].

It is necessary to underline that depending on the specific prebiotic, variety in the diet, the health status of the host-specific composition of the microbiota, and the individual transit time of the gut, levels of the produced SCFAs usually can reach concentrations of 50 to 200 mmol/kg of luminal content in the large intestine [62], where, acetate, propionate, and butyrate are in a proportion of 3:1:1 [63]. The complexity in the production of SCFAs is associated with the specific metabolic pathways involving intermediate metabolites, including pyruvate, succinate, lactate, 1,2-propanediol, and acetyl-coenzyme A (CoA) [64]. In these processes, some additional metabolites, including ethanol, propanol, and 2,3-butanediol, can be produced as terminal products of carbohydrate fermentation and, in some cases, even in low concentrations, cannot be neglected [64].

During fermentation, the production of specific gases as the end metabolic products (hydrogen—H_2_, carbon dioxide—CO_2_, and sulfur oxides—SOx) can be formed. Hydrogen is often necessary for the cycling of *NAD*^+^/NADH and can be further used by methanogens and sulfate reducers to produce CH_4_ (methane) and H_2_S (hydrogen sulfide). The processes of anaerobic respiration, through the membrane electron transport chain, increase the capacity to use the produced H_2_ and CO_2_ by reductive acetogen and methanogen microorganisms, respectively, and SO_4_^2−^ by sulfate-reducing microorganisms, as electron acceptors [64,65]. Most intestinal gases are absorbed into the bloodstream and removed via the lungs; however, depending on the volume of gas production, it could distend the colonic wall and affect the speed of material transition through the colon [66]. It also represents an undesirable effect of fermentable non-digestible polysaccharides, known as bloating, one characteristic prebiotics should avoid since the output must only benefit the subjects.

After the gut’s microbial populations produce acetate and propionate, they are further absorbed by the colonic wall. In the following, acetate and propionate enter the portal blood compartment and are preferentially metabolized in the liver. In the liver, they are involved in the hepatic biosynthesis pathways, including gluconeogenesis, cholesterol, and long-chain fatty acids synthesis, and as a result, contribute to the whole host metabolism [67].

Short-chain fatty acids are involved in microbial-signaling processes, where are recognized by G-protein-coupled receptors, GPR41 (also known as Free fatty acid receptor 2 (FFA2)), GPR43 (also known as Free fatty acid receptor 3 (FFA3)), GPR109A (also known as Hydroxycarboxylic acid receptor 2 (HCA2)), and Olfactory receptor 78 (Olfr78) [68,69]. It is essential to underline that GPR41 and GPR43 are expressed by entero-endocrine cells where they can be directly associated with the activation of the production of glucagon-like peptide-1 (GLP-1) and the appetite-regulating hormone peptide YY (PYY) [70]. Moreover, the expression of these receptors is directly associated with the production and activations of specific immune cells, providing clear evidence for the beneficial roles of the SCFAs, especially those involving immune response and inflammation [71].

For some subjects with clinical disorders such as obesity and diabetes, the abundance of butyrate-producing species is lower than in healthy intestinal microbiota [72,73]. Moreover, the amount and variety of ingested dietary fibers were associated with the modulation of the relative abundances of taxa and microbial genes related to butyrate production. Furthermore, a low-fiber diet has been associated with the reduced relative abundances of specific butyrate-producing microbes in humans [74], non-human primates [75], and mice, an effect that is more pronounced from generation to generation [76].

## 6. Hydrolyzed Pectin and Oligosaccharides as Potential Prebiotics

How POS modulate gut microbiota depends on their structural characteristics, including the proportion of RG-I domains, DE, and overall conformation. Additionally, the side chains of pectin not only augment its prebiotic effects but directly regulate IL-6 production in intestinal host cells [46]. Table 1 and Table 2 show the main in vitro, in vivo, and clinical studies on modified pectins, their modification process, the structures they obtained, and the relevant results. Only studies done with pectin oligosaccharides and/or pectin fragments with up to 10 years of publication were considered.

Current literature suggests that while promising preliminary data exist on the immunomodulatory effects of non-digestible oligosaccharides, including pectin, more extensive human studies are needed to confirm these findings. In vitro models show that prebiotics, such as FOS, inulin, and GOS, can influence immunity by modulating the interaction between residual microbiota and Toll-like receptors on monocytes, macrophages, and intestinal epithelial cells. Additionally, prebiotics may beneficially affect immune responses by specifically modulating cytokine production and immune cell maturation. Consistent with these findings, animal models and human supplementation trials have demonstrated that certain prebiotics (including inulin and lactulose) may help reduce intestinal inflammation and alleviate symptoms of inflammatory bowel disease (IBD). However, the precise mechanisms of action remain unclear, and further studies are required to explore a broader range of prebiotics [90].

Pectins have been implicated in promoting the generation of peripheral regulatory T cells (Tregs) through epigenetic modulation and suppressing the inflammatory functions of dendritic cells (DCs) via transcriptional regulation. Moreover, pectins appear to alter the ratio of Firmicutes to Bacteroidetes in the gut and lung microbiota, thereby increasing the concentrations of short-chain fatty acids (SCFAs) in feces and serum and reducing airway inflammation by inhibiting DC function [31].

Furthermore, Weber et al. [91] proposed that pectins play a crucial role in modulating gut health, directly contributing to postprandial glucose regulation, and are associated with maintaining healthy blood cholesterol levels. They emphasize the need for further research to understand better the relationship between pectin’s structure and its health benefits, highlighting the importance of a more accurate characterization of its nature, physicochemical properties, and molecular composition, such as the degree of esterification, molecular weight, branching, viscosity, gel formation, and solubility [91].

Some studies have identified potential prebiotic properties of POS by using different types of pectin and different study models—from in vitro assays to animal models, in a clear outcome of robust pre-clinical tests. The stratification using specific conditions for extraction and modification was necessary to separate and indicate discussions that applied the structure-function approach, which is essential when studying pectin [92,93]. All of the studies shown in Table 1 and Table 2 demonstrated the enhancement of the bioactivity of POS generated by the applied methods, focusing on improving gut microbiota growth or other biological processes.

One example is a POS obtained from galacturonic acid and rhamnose hydrolyzed from pectin-rich by-products. The study showed increased proliferation of *B. animalis* and production of SCFAs such as acetic and propionic acids, which are relevant for gut health. Other oligosaccharides extracted from jaboticaba were studied by hydrolysis of starch with specific enzymes and pectin treatment with an oxidizing reagent. These results showed the inhibition of galectin-3-driven hemagglutination. They lowered the viability of colorectal cancer cells, which signals the use of these oligosaccharides in cancer prevention, but with no citation of a potential microbiota modulation [77]. In another study using fermentation assays and bacterial growth that employed POS and sugar beet pulp (SBP) or discarded red beetroot (DRB), POS, upon enzymatic hydrolysis, resulted in the most substantial increases of *Lbs. rhamnosus* growth rates, SCFA production, as well as lactate production, thus indicating its high prebiotic potential [11]. Similarly, in the case of okra pectin degradation by the action of pectinase and using an in vitro fermentation approach, the hydrolysis led to a significant elevation of SCFA-producing bacteria such as bifidobacteria and clostridia while decreasing the relative abundance of pathogenic *Escherichia-Shigella* and hence leading to healthy gut microbiota [78].

Apple pectin fractions obtained through acid extraction with citric acid and ultrasound treatment led to the growth of *Akkermansia* spp. and *Blautia* spp.; beneficial gut microbes in an in vitro digestion approach. The beneficial results occurred only with apple-derived pomace and pectin, not other non-pectic substrates [79]. Another commercial prebiotic preparation of apple pomace resulted in the induction of bifidobacteria growth in a child’s feces and the production of SCFAs, further establishing the beneficial role of POS in gut health [80]. The research on POS from *Actinidia arguta* used hydrolysis and enzymatic degradation with ultrasound, and it observed that prebiotic activity increased with the degree of branching of oligosaccharides in an in vitro fermentation approach, a breakthrough analysis since pectin-derived oligosaccharides branches are challenging to study chemically [81].

A study by Sundaram et al. [94] reported that citrus pectin, combined with omega-3 polyunsaturated fatty acids and milk-derived exosomes, contributed to maintaining intestinal barrier integrity and positively modulated immune responses in livestock and poultry. The study also emphasized the potential of citrus pectin to enhance overall health by exhibiting anti-inflammatory, antimicrobial, and prebiotic properties [94]. Similarly, Tang and De Vos (2023) [95] demonstrated that different pectins, particularly those derived from citrus, can positively influence the gut’s immune barrier. These pectins support beneficial gut bacteria while strengthening the mucosal and epithelial layers, both critical for proper immune function [95].

Weber et al. [91] suggested that pectins may effectively modulate immune responses, enhance gut health and promote human SCFA production. However, the authors highlighted a significant gap in research concerning the relationship between pectin’s structure and its specific health outcomes. Donadio et al. [29] discussed the dual immunomodulatory effects of pectins, explaining that they can act directly on the intestinal barrier by interacting with Toll-like receptors (TLRs), thereby reducing inflammation.

Additionally, pectins can indirectly influence the immune system by altering the composition and diversity of gut microbiota through metabolic activity, leading to increased SCFA production. These metabolites, particularly SCFAs, are crucial in modulating gut health and immune function [96]. Thus, the modulation of gut microbiota and subsequent SCFA production are key mechanisms by which pectins exert their immune-regulatory effects.

**Table 2:** Recent in vivo studies and clinical trials with pectic oligosaccharides (POS) as potential prebiotics *.

Type of Pectin	Type of Study	Extraction and Modification Characteristics	Main Biological Results
A combination of short-chain GOS, long-chain FOS, and pectin-derived acidic oligosaccharides (pAOs)	Groups with 21 suckling rats, infected by rotavirus, ingested the combination for 18 days	-	Showed a softer consistency of fecal samples, sometimes considered diarrheic, viral shedding was greatly reduced, and blocking assay indicated a certain inhibition in detecting the virus [97]
Pectin-derived acidic oligosaccharides (pAOs)	C57BL/6 mice for 5 weeks consumed 5% of pAOs diet and were chronically infected by *Pseudomonas aeruginosa*	-	The increased butyric acid concentration in feces, is related to better control of the inflammatory response and a decrease in bacterial load [98]
POS from commercial pectin	In vivo model C57BL/6J mice, with n = 26 divided into 4 groups for 12 weeks of intervention	Enzymatic hydrolysis	POS supplementation in HFD-fed mice decreased body weight (*p* < 0.01), improved glucose tolerance (*p* < 0.001), reduced-fat accumulation (*p* < 0.0001) and hepatic steatosis protects the intestinal barrier and reduces pro-inflammatory cytokine levels [99]
A combination of short-chain GOS, long-chain FOS and low-viscosity pectin with and without *Bifidobacterium breve*	Male BALB/cByJ mice (20 per group) received 16 intra-gastric doses (25 mg) for 15 days	Low-viscosity Pectin is a 9:1:2 ratio	Neutrophilic lung inflammation coexisted with attenuated levels of fecal SCFA. The beneficial effects of the synbiotic mixture of *B. breve* M16-V and GOS:FOS:lvPectin on lung health are associated with enhanced levels of SCFAs [100]
POS from citrus pectin	10 Female BALB/c for 42 days with a daily dose of 2,5 g POS/kg body weight	POS 2-10 mer	Mice fed POS increase IgG, slkA, IgA, IL-12 and decrease the concentration of pro-inflammatory cytokines IL-5, IL-6, IL-13 and IL-17 in lung and blood serum after Poly I: C stimulation [101]
POS from citrus peel pectin	6 groups of 8 male BALB/c mice each for 4 weeks of administration	Hydrogen peroxide under alkaline conditions	Intestinal microbiota composition changed with a higher fecal concentration of acetate, which leads to an increase in the levels of fecal secretory immunoglobulin A and serum IgC [102]
Pectin-derived polygalacturonic acid (POAS)	24 male C57BL/6 obese mice divided into 4 groups for 24 weeks	Enzymatic hydrolysis	POAS promoted SCFAs production and alleviated the gut microbiota dysbiosis caused by a high-fat diet [103]
Acidic oligosaccharides derived from pectin (pAOs)	Male BALB/c mice into 2 groups fed with 5% of pAOs for 5 weeks for 35 days	-	Modified the intestinal microbiota by stimulating the growth of species involved in immunity development like *Bifidobacterium* sp., *Sutturella wadsworthia* and *Clostridium* cluster VIVa organisms [104]
Fragments of pectin (ALP2)	ICR mice (male) were divided into 8 groups (n = 10) and administered the dose for 7 days	Enzymatic hydrolysis (ALP2-E) and acid enzymatic hydrolysis (ALP2-A)	ALP2-A could alleviate the constipation symptoms of mice while ALP2-E had better intestinal regulation activity [105]
Partially hydrolyzed pectin (PHP) from passion fruit peel	40 weaned male Wistar rats (n = 8) received diets with PHP in crescent levels for 38 days	Hydrolysis	Fecal pH decreased with consumption of PHP 1,00% and the production of propionic and butyric acids in the cecum was higher in treatments with PHP [106]
Pectin oligosaccharides (pec-oli)	C57BL/6J (B6) mice were assigned to 3 groups, the pec-oli group received a daily dose of 0.8 g/kg body weight	Chemical degradation method	The pec-oligo microbiome causes elevated IgA in a T cell-dependent fashion, and induced bacteria promote the IgA-high phenotype in the small intestine [107]
POS	Male C57BL/6J mice were divided into 3 groups of 6 each and induced colitis, fed with different diets for 3 weeks	Enzymatic hydrolysis	POS promoted colonic epithelial barrier integrity, reduced inflammatory cytokines and regulated the Treg/th17 balance [108]
Short-chain GOS, long-chain FOS and pectin hydrolysate-derived acidic oligosaccharides	In a clinical trial with 57 healthy adults, the use of 15 or 30 g/d for 12 weeks	Hydrolysis	Increased bifidobacteria, decreased *Clostridium coccoides/Eubacterium rectale* cluster. Increased NK cell activity, and reduced activation of CD14, and CD25 [109]

* Studies older than 10 years were excluded from the table.

In animal models with mice (Table 2), POS produced from commercial citrus pectin was tested for 12 weeks. It showed that mice on a high-fat diet supplemented with POS had reduced body weight, improved glucose tolerance, and lowered fat accumulation. This same study also delineated that POS protected the intestinal barrier and reduced levels of pro-inflammatory cytokines, showing its anti-inflammatory benefits along with metabolic ones [83]. At least just one clinical trial was found. The clinical study in which short-chain GOS, long-chain FOS, and pectin-derived acidic oligosaccharides were given to healthy subjects showed increased bifidobacteria, a reduction in *Cl. coccoides*/*Eub. rectale*, improved NK cell activity with a decrease in the activation of immune markers, presenting bifidobacterial gut microbiota and immune benefits [82].

These findings suggest that different sources and extraction methods of pectin might have an essential impact on shaping the biological effects of POS. Tailored hydrolysis and enzymatic treatments often enhance the prebiotic potential, and the results have repeatedly shown improvements in gut microbiota composition and SCFA production, enabling systemic health benefits across models and indeed demonstrating the potential prebiotic effects of POS.

Due to the biological complexity of the microbiota and all the factors that interfere with it, establishing a direct relationship between the prebiotic and the benefit to the host, even with biomolecular techniques, is a great challenge. Added to this is the fact that pectin is also a complex molecule, which, whether native or modified, confers benefits that are already well-established in the literature. However, no matter how simple the modification technique used, many studies must be carried out to determine the modification parameters (e.g., size of the molecule, chemical structure, solubility, etc.) and the safety of its consumption (e.g., microbiological tests, toxicity, etc.). There are facts that justify the absence of clinical studies in more advanced stages.

It is also worth noting that even among already established prebiotics, there are some critical points to be considered in studies, such as the use of standardized protocols in terms of compounds (uniformity in the chemical structure for some classes, as well as a similar degree of polymerization), doses, methods of administration (with food or as spheres), duration of clinical trials and type of supplementation (alone or with probiotic microorganisms) [110].

As previously demonstrated, sufficient data in literature supports modified pectins as potential prebiotics. In this sense, the selective modulation of the microbiota, the production of metabolites, mainly SCFA and their beneficial effects, as well as the increase in the abundance of probiotic bacteria, the inhibition of pathogen growth, the decrease in intestinal pH, which helps in modulation and, finally, the undesirable effect of gas production and bloating effect, are also highlighted. However, many more shreds of evidence on humans must be determined since just one clinical trial has linked POS ingestion with increased human health [82].

## 7. Clinical Trials and Prebiotics: Current Knowledge and Future Perspectives

The most well-known prebiotics are fructooligosaccharides (FOS), galactooligosaccharides (GOS), inulin and lactulose. Even though some prebiotics are well established, in January 2024, 595 clinical studies with prebiotics were registered, of which 362 were complete, but only 16 were published [110]. Although some prebiotics have shown promise in early-stage animal and human studies, double-blind, randomized, and placebo-controlled trials (RCTs) remain challenging. Obstacles include variability in gut microbiota between individuals, difficulty in standardizing dosages, and the complexity of measuring long-term health outcomes. As they are essential for establishing a prebiotic, Table 3 contains some examples of studies carried out with already consolidated prebiotics.

Double-blind, randomized, and placebo-controlled clinical trials (RCTs) are considered the gold standard for clinical research, but they have their challenges, especially when studying complex biological interventions like prebiotics. Eliminating bias is probably the greatest asset of a double-blind study because neither the participants nor the researchers know who receives the intervention or the placebo. Randomization, where the assignment is random, is especially important in prebiotic studies, where individual differences in gut microbiota can influence the results. The placebo is essential to prove that the intervention, rather than some unrelated factor, causes the observed effects [118].

As already mentioned, due to the complexity of the microbiota, obtaining a direct link to host benefits is a major challenge. This is why it is necessary to associate it with advanced techniques such as microbiome sequencing and metabolomics, which can make studies expensive and time-consuming. Even so, interindividual variability must be considered since establishing standards concerning the microbiota is not always possible. The time factor must also be considered since the biological effects are long-term, which increases the cost and complexity of the study, in addition to often reducing the number of volunteers [118].

## 8. Final Thoughts

When ingested as dietary fiber, galactan, for example, is fermented in the large intestine, where gut bacteria initiate its breakdown into galactooligosaccharides (GOS) using extracellular GH53 endo-β-1,4-galactanase. At this stage, GOS exhibits structural similarities to specific oligosaccharides commonly found in the market and one of the major commercial prebiotic types [49]. Humans do not digest native pectin with endogenous enzymes to break this complex biomolecule. However, certain bacteria of the gut (e.g., *Bacteroides*, *Bifidobacterium*, *Clostridium*, *Erwinia*, *Escherichia*, and *Eubacterium* strains) can use pectin as substrates for fermentation [39]. The use of artificial techniques to modify pectin, create new pectin-derived oligosaccharides, and enhance its benefits in GIT is well-established for in vitro and in vivo approaches, with promising results. In addition, these methods provide a valuable alternative for repurposing agro-industrial waste.

Nevertheless, establishing a direct link between the modified pectins and the resulting health benefits remains challenging due to the complexity of the human microbiome. Even with advanced biomolecular techniques that offer precise insights, a comprehensive understanding of the mechanisms of action for potential prebiotics still requires further investigation, mainly through clinical trials with larger and more representative sample sizes.

## Figures and Tables

**Table 1:** Recent in vitro studies with pectic oligosaccharides (POS) as potential prebiotics *.

Type of Pectin	Type of Study	Extraction and Modification Characteristics	Main Biological Results
POS from apple pomace	Caco-2 Cell Culture	Mild acid hydrolysis combined with enzymatic hydrolysis	Stimulation of lactic acid bacteria adhesion to the human epithelial cells and the fecal bacteria and pathogens showed much weaker adhesion to intestinal cells in the presence of all tested [77]
Oligosaccharides from jaboticaba	HCT116 cell culture	Hydrolysis of starch with amylase and protease	Inhibition of Gal-3hemagglutination and colorectal cancer cell viability [78]
POS + Sugar beet pulp (SBP) or discarded red beetroot (DRB)	Fermentation assays and bacterial growth	POS production by enzymatic hydrolysis	The highest maximum growth rate for *Lacticaseibacillus rhamnosus*, SCFA, and lactate production [11]
POS Galacturonic acid, rhamnose	In vitro fermentation	Extraction from pectin-rich food by-products followed by hydrolysis	Higher abundance of *Bifidobacterium animalis* TISTR 2195. Formation of acetic and propionic acid [79]
Pectin from okra	In vitro fermentation	Pectinase hydrolysis	The highest level in SCFA-producing bacteria (*Bifidobacterium* sp. And *Clostridium* sp.) and SCFA beneficial molecular, along with the low relative abundance of pathogenic bacteria *Escherichia-Shigella* [80]
Pectin fractions from apple	In vitro digestion	Acid extraction with citric acid in an ultrasound bath	The growth of *Akkermansia* sp., *Blautia* sp., *Eubacterium eligens* group, *Intestinimonas* sp., and *Succinivibrio* sp. only occurred with pomace and pectin derived from the tested by-products, not other non-pectic prebiotics/substrates [81]
Prebiotic preparation of apple pomace	In vitro fermentation	Method development by Agros Nova Company	POS highly stimulated *Bifidobacterium* sp. on a 7-year-old child. SCFAs produced were acetic and propionic acid [82]
POS from fruit *Actinidia arguta*	In vitro fermentation	Hydrolysis and ultrasound enzymatic degradation	The prebiotic activity of POS was positively correlated with the branching degree [83]
Pectins from pomelo *(Citrus maxima)*	In vitro fermentation	Subcritical water extraction and enzymatic modification	Heightened the fermentability of pectins and altered uronic acid utilization patterns, expediting the production of SCFAs. Induced changes in microbial composition, elevating the relative abundance of *Bacteroides* sp. while suppressing potential pathogens like the *Escherichia-Shigella* group [84]
Pectic oligosaccharides from sugar beet pulp (DP2-13)	In vitro fermentability assessment and fluorescent in situ hybridization (FISH)	Hydrothermal treatment followed by enzyme treatment	Increase *Bifidobacteria* spp., and lactobacilli, *Faecalibacterium* and *Roseburia* also increased their counts with all the substrates (especially with POS). The highest concentrations of organic acids were observed in media containing oligosaccharides [85]
POS from orange peel wastes	In vitro fermentation and fluorescent in situ hybridization (FISH)	Hydrothermal treatment	POS boosted bifidobacteria and lactobacilli, POS fermentation was similar to FOS fermentation, but pectin reduced butyrate generation [86]
Pectin-derived galacturonic acid oligosaccharides (GalA-OS) from lemon peels	In vitro batch fermentation using Simulator of Human Intestinal Microbial Ecosystem (SHIME)	Enzyme treatment	Induced the production of acetate, butyrate and propionate. Stimulates specifically *Clostridium butyricum* [87]
POS	In vitro gut Microbiota fermentation	Chemical controllable degradation method	POS increased the abundance of the cholesterol-related bacterial groups *Bacteroides*, *Bifidobacterium* and lactobacilli, while decreasing *E. coli* and *Enterococcus* spp. [88]
Pectin polysaccharide (PEC), partially hydrolyzed pectin (PPH) and pectin oligosaccharide (POS)	In vitro fermentations using the CoMiniGut model with healthy elderly and young adults’ feces	Hydrolysis	No significant differences in SCFAs production were found among the pectins. Pectins boosted various bacterial groups differently from the reference (inulin) [89]

* Studies older than 10 years were excluded from the table.

**Table 3:** Examples of clinical trials using prebiotics *.

Prebiotic	Trial Type	Intervention	Main Biological Results	Reference
β-fructans inulin plus FOS	Dual arm, exploratory study	15 g day^−1^ or 7.5 g day^−1^ for 9 weeks in 25 ulcerative colitis patients	The high fructan dose led to an increased *Bifidobacteriaceae* and *Lachnospiraceae* abundance but these shifts were not correlated with improved disease scores.	[111]
GOS	Single arm, open label	2,8 g day^−1^ for 6 weeks with 18 ulcerative colitis patients	Prebiotics did not lower clinical scores or inflammation but normalized stools. *Bifidobacterium* and *Christensenellaceae* proportions only increased in patients with less active diseases, indicating that the prebiotic effect may depend on disease activity.	[112]
FOS	Parallel, placebo-controlled, randomized and double-blind	5 d/day for 4 weeks with 79 patients with irritable bowel syndrome and rectal hypersensitivity	Short-chain FOS significantly reduced anxiety scores and increased fecal *Bifidobacteria* without modifying other bacterial groups	[113]
FOS	Randomized, double-blind, placebo-controlled parallel	6,9 or 12 d/day for 4 weeks with 36 infants with constipation	FOS was associated with reduced bowel transit time and higher counts of the genus *Bifidobacterium* in the stool	[114]
Inulin	Randomized, single-blinded, multicentric, placebo-controlled trial	16 g/day for 3 months with 150 obese patients	Inulin-enriched diet was able to promote weight loss in obese patients, the treatment efficiency is related to gut microbiota characteristics	[115]
Inulin	Randomized, double-blind, placebo-controlled crossover trial	10 g/day for 12 weeks with 16 continuous ambulatory peritoneal dialysis participants	Inulin-type prebiotics were promising therapeutic candidates for reducing serum uric acid levels in renal failure patients, and this lowering effect was attributed to intestinal microbial degradation of uric acid	[116]
Inulin	Cross-sectional case-control study integrating clinical variables	10 g/day for 3 months with 49 patients newly diagnosed pre/diabetes, 66 metabolically healthy overweight/obese, and 32 healthy lean volunteers	The inulin addition led to a general enhancement in glycemic indicators	[117]

* Studies older than 10 years were excluded from the table.

## Data Availability

No new data were created or analyzed in this study. Data sharing does not apply to this article.
